# The novel TRPA1 antagonist BI01305834 inhibits ovalbumin-induced bronchoconstriction in guinea pigs

**DOI:** 10.1186/s12931-021-01638-7

**Published:** 2021-02-08

**Authors:** Mariska P. M. van den Berg, Susan Nijboer-Brinksma, I. Sophie T. Bos, Maarten van den Berge, David Lamb, Martijn van Faassen, Ido P. Kema, Reinoud Gosens, Loes E. M. Kistemaker

**Affiliations:** 1 Department of Molecular Pharmacology, University of Groningen, Antonius Deusinglaan 1, 9713 AV Groningen, The Netherlands; 2 Groningen Research Institute for Asthma and COPD (GRIAC), University of Groningen, University Medical Center Groningen, Groningen, The Netherlands; 3 Department of Pulmonary Diseases, University of Groningen, University Medical Center Groningen, Groningen, The Netherlands; 4 Immunology + Respiratory, Boehringer Ingelheim Pharma GmbH & Co. KG, Biberach an der Riss, Germany; 5 Department of Laboratory Medicine, University Medical Centre Groningen, University of Groningen, Groningen, The Netherlands

**Keywords:** Asthma, Airway hyperresponsiveness, Airway smooth muscle, Mast cell, Sensory neuron

## Abstract

**Background:**

Asthma is a chronic respiratory disease in which the nervous system plays a central role. Sensory nerve activation, amongst others via Transient Receptor Potential Ankyrin 1 (TRPA1) channels, contributes to asthma characteristics including cough, bronchoconstriction, mucus secretion, airway hyperresponsiveness (AHR) and inflammation. In the current study, we evaluated the efficacy of the novel TRPA1 antagonist BI01305834 against AHR and inflammation in guinea-pig models of asthma.

**Methods:**

First, a pilot study was performed in a guinea-pig model of allergic asthma to find the optimal dose of BI01305834. Next, the effect of BI01305834 on (1) AHR to inhaled histamine after the early and late asthmatic reaction (EAR and LAR), (2) magnitude of EAR and LAR and (3) airway inflammation was assessed. Precision-cut lung slices and trachea strips were used to investigate the bronchoprotective and bronchodilating-effect of BI01305834. Statistical evaluation of differences of in vivo data was performed using a Mann–Whitney U test or One-way nonparametric Kruskal–Wallis ANOVA, for ex vivo data One- or Two-way ANOVA was used, all with Dunnett’s post-hoc test where appropriate.

**Results:**

A dose of 1 mg/kg BI01305834 was selected based on AHR and exposure data in blood samples from the pilot study. In the subsequent study, 1 mg/kg BI01305834 inhibited AHR after the EAR, and the development of EAR and LAR elicited by ovalbumin in ovalbumin-sensitized guinea pigs. BI01305834 did not inhibit allergen-induced total and differential cells in the lavage fluid and interleukin-13 gene expression in lung homogenates. Furthermore, BI01305834 was able to inhibit allergen and histamine-induced airway narrowing in guinea-pig lung slices, without affecting histamine release, and reverse allergen-induced bronchoconstriction in guinea-pig trachea strips.

**Conclusions:**

TRPA1 inhibition protects against AHR and the EAR and LAR in vivo and allergen and histamine-induced airway narrowing ex vivo, and reverses allergen-induced bronchoconstriction independently of inflammation. This effect was partially dependent upon histamine, suggesting a neuronal and possible non-neuronal role for TRPA1 in allergen-induced bronchoconstriction.

## Background

Asthma affects over 300 million people worldwide [1]. Characteristics of asthma include airway hyperresponsiveness to specific and non-specific stimuli, obstruction of airflow and airway inflammation [2]. Current therapy is focused on short-term alleviation of bronchospasms with inhaled bronchodilators, counteracting inflammation with inhaled corticosteroids on the long-term, or a combination of both treatments [3]. Even though existing treatments are suitable for many patients, there is a substantial group of asthmatic patients with unmet medical needs as, despite being prescribed with high-dose anti-inflammatory drugs and bronchodilators, their disease remains difficult to control [4]. This often leads to symptom specific-treatment, deconstructing the disease into treatable elements such as airway inflammation, bronchial hyperresponsiveness and cough reflex hypersensitivity, which each ask for a different therapeutic approach [5]. To aid these patients there is a large interest in the development of biologicals, aimed at inhibiting among others immunoglobulin E (IgE) or T-helper 2-specific cytokines [6]. However, as it has become clear that not only immune mechanisms are pivotal in asthma pathology, research into other possible key players is of great importance.

The peripheral nervous system is thought to play a central role in asthma, as smooth muscle contraction is largely controlled by the sensory and motor neurons that innervate the lungs and airways [7–9], and long-acting anticholinergics are effective bronchodilators in asthma [10]. Furthermore, increasing evidence suggests that there is plasticity of the nervous system in asthma, reflected by increased excitability of neurons and even outgrowth of neurons [11], as shown recently for sensory and cholinergic neurons in biopsies from asthma patients compared to healthy subjects [12, 13] Furthermore, in asthma, there is increased release of peptide neurotransmitters, e.g. substance P and neurokinin A, which can contribute to neurogenic inflammation [14]. Sensory nerve activation leads to respiratory symptoms such as reflex cough, bronchoconstriction and mucus secretion [14]. Induction of these respiratory symptoms could be attributed to the activation of specific receptors that are positioned at nerve terminals, including Transient Receptor Potential Ankyrin 1 (TRPA1) channels [15].

TRPA1 channels are located at sensory nerves, predominantly on C-fibers. In addition, non-neuronal cells including airway inflammatory cells, smooth muscle cells, epithelial cells and fibroblasts were shown to express TRPA1 [15–19]. TRPA1 channels are identified as important sensors of noxious stimuli and tissue damage [15]. (1) Spicy food extracts, such as garlic, cinnamon and mustard oil, (2) environmental irritants present in cigarette smoke, vehicle exhaust and air pollution, and (3) radical oxygen species are among the known agonists of TRPA1. Activation of TRPA1 channels is implicated in cough, as triggering of TRPA1 channels leads to the activation of bronchopulmonary C-fibers in rodent animal models and evokes cough in guinea pigs [15, 17–21]. This was confirmed in healthy human subjects, in whom activation of TRPA1 results in cough and triggering of TRPA1 channels stimulates isolated human vagal tissue [17]. In asthma, TRPA1 may be involved in several aspects of the disease. TRPA1 activation contributes to the development of airway hyperreactivity (AHR) in asthma [22] and to the late asthmatic response (LAR) [23]. Knock-out or inhibition of TRPA1 channels inhibits neuropeptide release and AHR in ovalbumin (OA)-challenged mice [24]. The same study showed inhibition of allergen-induced leukocyte infiltration in the airways and a decrease in cytokine production, indicating a role of TRPA1 in allergen-driven asthmatic airway inflammation. Furthermore, a role of TRPA1 in airway inflammation mediated via non-neuronal cells including human airway epithelial, smooth muscle cells and fibroblasts is also shown [25]. In addition, inflammatory mediators can directly activate TRPA1 [26, 27] and TRPA1 inhibition may prevent mast cell degranulation [28]. Finally, Gallo et al*.* showed associations of TRPA1 gene variants with childhood asthma [29]. Together, this suggests that TPRA1 inhibition represents an interesting therapeutic target for asthma, as it may combine bronchoprotective, antitussive and anti-inflammatory effects.

Even though several patents have been filled for novel TRPA1 antagonists in respiratory disease, there are currently no clinically approved TRPA1 inhibitors [30, 31]. In this study, we evaluated the efficacy of the novel TRPA1 antagonist BI01305834, and investigated how this antagonist could alleviate asthma symptoms in guinea-pig models of allergic asthma, via neuronal and non-neuronal pathways. We demonstrate that BI01305834 protects against AHR and the early asthmatic response (EAR) and LAR in an acute OA-induced in vivo model, against allergen and histamine-induced airway narrowing in precision cut lung slices, and is able to partially reverse allergen-induced bronchoconstriction in tracheal strips ex vivo.

## Methods

### In vitro* IC*_*50*_* assessment*

HEK cells overexpressing human TRPA1 were pre-incubated for 2 h with Calcium 6 dye (Molecular Devices) in HBSS [+ CaCl_2_/MgCl_2_] + 20 mM HEPES + 0.1% BSA. BI01305834 was applied 30 min prior to stimulation in DMSO (final concentration 0.1%) and cells stimulated with 5 µM supercinnamaldehyde. Calcium flux was measured as fluorescence using FLIPR. Concentration response curves were plotted and IC_50_-values calculated using GraphPad Prism.

### Compliance with requirements for studies using animals

Male Dunkin-Hartley guinea pigs (Envigo, NL) weighing approximately 500 g and 650–850 g were used in this study for in vivo and ex vivo experiments respectively. The animals were group-housed in individual cages in climate-controlled animal quarters, and given water and food ad libitum, while a 12-h on/12-h off light cycle was maintained. In airway pharmacology perspective, guinea pigs are superior experimental animals compared to mice and rat as they better resemble human airway physiology [32]. The specific model used in this study allows for measurements on conscious, unrestrained animals and importantly, it also enables the monitoring of the full EAR and LAR [33]. All protocols described were approved by the University of Groningen Committee for Animal Experimentation (license AVD105002016492 and AVD10500201581).

### *Experimental protocol guinea-pig *in vivo* studies*

**Fig. 1:**
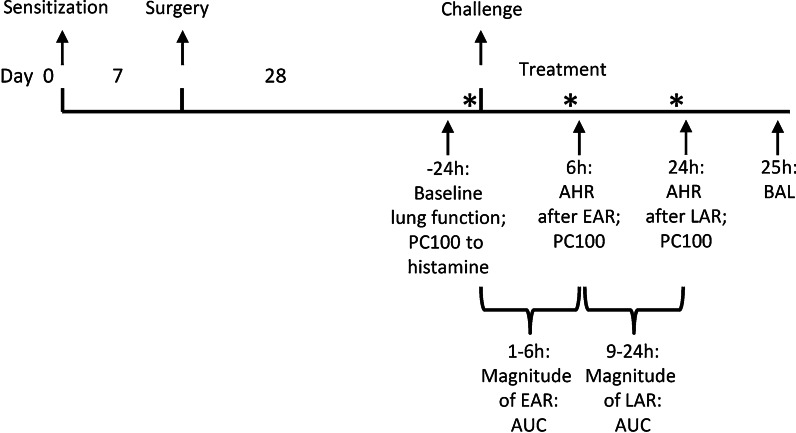
Overview of the experimental acute asthma protocol. *: Animals were treated 30 min before ovalbumin challenge and/or the early asthmatic reaction (EAR) and/or the late asthmatic reaction (LAR). Airway hyperresponsiveness in response to histamine was assessed at the time of EAR and LAR. A bronchoalveolar lavage (BAL) was performed 25 h after the ovalbumin challenge

The experimental protocol is depicted in Fig. 1.

Animals were actively IgE sensitized to OA (Sigma-Aldrich) by injecting 1.0 ml of an allergen solution containing 100 μg.ml^−1^ OA and 100 mg.ml^−1^ Al(OH)_3_ in saline. Of this 1.0 ml, 0.5 ml was injected i.p., while another 0.5 ml was divided over seven s.c. injection sites in the proximity of lymph nodes in the paws, lumbar regions and neck, as described previously [34]. One week after sensitization, the animals for the in vivo study were surgically provided with a balloon catheter in the thoracic cavity, as outlined below. Animals were treated via oral gavage with the TRPA1 antagonist BI01305834 on t = -0.5 h before ovalbumin challenge, t = 5.5 h after challenge and t = 23.5 h after challenge (Fig. 1). These time points were chosen based on previous assessments in this model, showing that AHR after the EAR is best measured at t = 6 h and AHR after the LAR is best measured at t = 24 h [33]. To allow for target binding, the TRPA1 antagonist was administered 30 min prior to these measurement time points.

In the pilot study, animals received 0.1, 1 or 10 mg.kg^−1^ BI01305834. In the main study, animals were treated with 1 mg.kg^−1^ BI01305834. In the vehicle-treated groups, 7 animals were included in the saline-challenged group and 11 in the OA-challenged group. In the groups treated with BI01305834, 7 animals were included in the saline-challenged groups and 13 in the OA-challenged group. Group sizes were initially designed to be equal in size and calculated using airway eosinophilia as the primary read-out parameter, with alpha = 0.05, sigma = 20 and mu1-mu2 = 30 (based on known parameters from previous studies). However, some animals were lost during surgery or suffered from extensive inflammation as a result of the surgery, and therefore had to be excluded from analysis. Vehicle/OA and BI01305834/OA groups were complimented with animals from pilot study to increase power for statistical analysis.

### Measurement of lung function

Animals underwent surgery to install an intrapleural balloon catheter to measure lung function and EAR and LAR. Lung function was assessed by online measurement of pleural pressure (P_pl_) under conscious and unrestrained conditions as described previously [33, 35]. Before and during surgery animals were anaesthetized by inhalation of a mixture of N_2_O (500 ml.min^−1^)/O_2_ (500 ml.min^−1^)/3–5% isoflurane. 0.015 mg.kg^−1^ buprenorphine was administered by intramuscular injection before surgery for epi- and postoperative analgesia.

In short, a small fluid-filled latex balloon-catheter was surgically implanted inside the pleural space. The free end of the cannula was driven subcutaneously to the neck of the animal, where it was permanently exposed. Via an external saline-filled cannula the intrapleural balloon-catheter was connected to a pressure transducer (TXX-R,Viggo-Spectramed, Bilthoven, Netherlands) and P_pl_ was continuously measured using an online computer system.

Using a combination of flow measurement with a pneumotachograph, implanted inside the trachea, and pressure measurement with the intrapleural balloon-catheter, it was previously shown that changes in P_pl_ are linearly related to changes in airway resistance and hence can be used as a sensitive index for allergen- and histamine-induced bronchoconstriction. In this way, airway function can be monitored continuously, while the animals are unaware of the measurements being taken and without the use of anaesthetics, as these function as TRPA1 agonists in guinea pigs [36].

Airway function measurements were carried out in a specially designed 9 l perspex cage in which the guinea pigs could move freely, as described previously [33]. A DeVilbiss nebulizer (type 646) driven by an airflow of 8 l.min^−1^ provided the aerosol with an output of 0.33 ml.min^−1^. All provocations were preceded by an adaptation period of 30 min, followed by a control provocation with saline, lasting 3 min.

### Habituation procedure

In order to reduce stress-induced effect on P_pl_-measurements, animals were first habituated to the experimental procedures in four training sessions. For the first session the animals underwent a control provocation with saline, lasting 3 min. During the second session, the animals’ intrapleural balloon-catheter was connected to the pressure transducer via the external saline-filled canula. Again, the animals underwent a control provocation. During the third and fourth training session, the animals were connected to the pressure transducer and histamine-provocations (PC_100_-measurements) were performed. In order to assess the airway reactivity to inhaled histamine, subsequent provocations with increasing concentration steps (6.25, 12.5, 25, 50, 75, 100, 125, 150, 175, 200 and 250 µg.ml^−1^) in saline were performed. Histamine provocations lasted maximally 3 min and were separated by 8 min intervals. Animals were challenged until P_pl_ was increased by more than 100% above baseline. The provocation concentration of histamine causing a 100% increase of P_pl_ (PC_100_) was derived by linear intrapolation of the concentration-P_pl_ curve and was used as an index for airway reactivity towards histamine. P_pl_ returned to baseline within 15 min after the last histamine provocation.

### Experimental procedure

In order to determine histamine responsiveness before allergen challenge, animals underwent a PC_100_-measurement to histamine 24 h before allergen challenge. Histamine provocations were performed as during training sessions.

Allergen provocations were performed by inhalation of OA in saline. The OA inhalation was discontinued when the first signs of respiratory distress were observed and an increase in P_pl_ of more than 100% was reached. First a 0.05% OA-solution in saline was used. When this dose was insufficient to induce respiratory distress within 3 min, a 0.1% OA solution in saline was used. OA-dose was calculated as exposure to µg OA x minute. After OA-challenge, animals were left connected to the pressure transducer to allow for measurement of lung function over time.

AHR induced by ovalbumin was assessed by exposing the guinea pigs to histamine, in line with the PC_100_-measurement to histamine 24 h before allergen challenge. AHR in response to histamine was measured 6 h after OA-challenge (after the EAR) and 24 h after OA-challenge (after the LAR). AHR was assessed as a ratio of histamine responsiveness pre- and post- ovalbumin challenge, both after the EAR and the LAR.

In addition to the airway responsiveness to histamine, the magnitude of the EAR and LAR was quantified, by assessing the area under the curve of the plearal pressure curve over time. This are measurements of baseline pleural pressure after ovalbumin exposure, measurements are taken every 5 min and the area under the curve from 1-6 h is calculated for the magnitude of EAR, and 9-24 h for the magnitude of LAR.

### Bronchoalveolar lavage

Animals were anaesthetized by inhalation of a mixture of N_2_O (500 ml.min^−1^)/O_2_ (500 ml.min^−1^)/ 5% isoflurane 25 h after OA challenge. Under terminal anaesthesia, blood samples were drawn by cardiac puncture into EDTA tubes. Blood samples were centrifuged at 4500 rpm for 5 min at 4 °C and the plasma aspirated and stored at -80 °C prior to analysis of BI01305834. The trachea was exposed and cannulated, and the lungs were lavaged gently using 5 ml of sterile saline at 37 °C, followed by three subsequent aliquots of 8 ml of saline. The recovered lavage samples were kept on ice, and centrifuged at 200 g for 10 min at 4 °C. The pellets were combined and resuspended to a final volume of 1.0 ml in phosphate-buffered saline (PBS) and total cell numbers were counted using a Casy Cell Counter (Model TT, Innovatis). For cytological examination, cytospin-preparations were stained with May-Grünwald and Giemsa stain. A cell differential was performed by counting at least 400 cells in duplicate in a blinded fashion, as described previously [33].

### PCR analysis

Lung homogenates were prepared by pulverizing the tissue under liquid nitrogen and total RNA was extracted using the NucleoSpin® RNA isolation kit (Macherey–Nagel, #740,955.250) according to the manufacturer's instructions. Total RNA concentrations were determined with a NanoDrop ND-1000 spectrophotometer. Equal amounts of total mRNA were then reverse transcribed (Promega, #A3500), and cDNA was subjected to real-time qPCR (Westburg, Leusden, The Netherlands). Real time qPCR was performed with SYBR green as the DNA binding dye (Roche, #04,913,914,001) on an Illumina Eco Real-Time PCR system, with denaturation at 94 °C for 30 s, annealing at 59 °C for 30 s and extension at 72 °C for 30 s for 40 cycles followed by 10 min at 72 °C. Real-time qPCR data were analysed using LinRegPCR analysis software [37] and GAPDH was used as a reference gene. Interleukin (IL)-4, IL-5 and IL-13 gene expression was assessed. Gene expression was presented as relative gene expression (N0) compared to GAPDH. The specific forward and reverse primers used are listed in Table 1.

**Table 1:** Primers used for qRT-PCR analysis

Gene	Primer sequence
Guinea pig IL-4	Forward – GGG TGC AAC CAC CAC ACC TT Reverse – TGG ACC CTG GGG ATC AGC AA
Guinea pig IL-5	Forward – TAC ACA AGG GGA AGC TCT GG Reverse – CCA GTT TGG TCT CAG CCT TC
Guinea pig IL-13	Forward – TCA CCC AGG ATC AGA AGA CC Reverse – CCA CCT CGA TCT TGG TGT CT
Guinea pig GAPDH	Forward – AGA TGG TGA AGG TCG GAG TG Reverse – GAC GAG CTT CCC ATT CTC AG

*IL* interleukin, *GAPDH* Glyceraldehyde 3-phosphate dehydrogenase

### Exposure to BI01305834

Blood serum concentrations of BI01305834 were determined by liquid chromatography / mass spectrometry using a 2.1 × 50 mm column, 5 µm, 100 Å at 40 °C, using a mobile phase of water containing 0.1% Formic Acid (A) and acetonitrile containing 0.1% Formic Acid (B) at a flow rate of 400 µL.min^−1^.

### Precision-cut lung slices

Precision-cut lung slices were prepared as described previously [38, 39] at least four weeks after sensitization. Sensitization was performed as described above. For the preparation of lung slices, animals were anaesthesized by intradermal injection of 100 mg.ml^−1^ ketamine containing 10^–4^ M isoproteranol and 5 mg.ml^−1^ diazepam. After loss of reflexes, animals were euthanized by intracardial injection with pentobarbital (Euthasol 20%, Produlab Pharma, Raamsdonksveer, the Netherlands) and exsanguinated. In order to fill the lungs with a low melting agarose solution (1.5% final concentration (Gerbu Biotechnik GmbH, Wieblingen, Germany) containing isoproterenol (1 µM) in CaCl_2_ (0.9 mM), MgSO_4_ (0.4 mM), KCl (2.7 mM), NaCl (58.2 mM), NaH_2_PO_4_ (0.6 mM), glucose (8.4 mM), NaHCO_3_ (13 mM), Hepes (12.6 mM), sodium pyruvate (0.5 mM), glutamine (1 mM), MEM-amino acids mixture (1:50), and MEM-vitamins mixture (1:100), pH = 7.2)) the trachea was cannulated. To solidify the agarose, ice was placed on the lungs for at least 30 min. Afterwards, the lungs were removed and placed on ice. Tissue cores with a diameter of 15 mm were prepared of the separate lobes. In cold medium consisting of CaCl_2_ (1.8 mM), MgSO_4_ (0.8 mM), KCl (5.4 mM), NaCl (116.4 mM), NaH_2_PO_4_ (1.2 mM), glucose (16.7 mM), NaHCO_3_ (26.1 mM), Hepes (25.2 mM), pH = 7.2 and isoproterenol (1 µM)) the cores were sliced into 500 µM slices with a tissue slicer (CompresstomeTM VF- 300 microtome, Precisionary Instruments, San Jose CA, USA). The lung slices were incubated in a humid atmosphere under 5% CO_2_/95% air at 37 °C. Lung slices were washed every 30 min, 3 times with medium containing isoproterenol and once with medium only to wash out the isoproterenol and kept overnight.

### Ex vivo* airway narrowing studies*

Lung slices were used for OA and histamine induced airway narrowing studies. In order to do this, lung slices were placed in 1 ml medium and fixed with a plastic ring. After 30 min pre-treatment with the TRPA1 antagonist BI01305834 (0.01, 0.1, 1 or 10 µM) or the vehicle (10% cyclodextrin) OA (10^–5^-10^2^ µg.ml^−1^) or histamine (10^–8^-10^–2^ M) dose–response curves were established. Using a microscope (Eclipse, TS100; Nikon) time-lapse images (1 frame per 2 s) of the lung slices were captured. The airway luminal area was quantified using image acquisition software (NIS-elements; Nikon) and expressed as percentage of basal area, as described previously [39, 40].

### Histamine determination

Lung slices were used for OA-induced histamine determinations, with separate slices for each condition individually. BI01305834 and vehicle pre-treated lung slices were challenged with OA (10 µg.ml^−1^) for 5 min. Untreated and unchallenged lung slices were used as a control for spontaneous histamine release. Supernatant was collected and lung slices were transferred to ice-cold acetic acid solution (0.08 M) and homogenized by sonication (10 s; 60 pulses)(Vibra Cell; Sonics, Newton, USA). Afterwards, the sonicated samples were centrifuged for 30 min at 15,000 rpm and 4 °C. Histamine levels of original supernatant and homogenized lung slice supernatant were assessed by liquid chromatography in combination with isotope dilution tandem mass spectrometry (LC–MS/MS). Histamine-d4 (Toronto Research Chemicals) was used as internal standard. Inter-assay imprecision (n = 20 days) was < 2.9% at three different levels (60, 986, 3873 nM, respectively) and limit of quantification was 3.0 nM. Histamine release was calculated as percentage of total histamine present in both supernatant and slice. Data of histamine released by BI01305834 pre-treated lung slices was normalized to histamine released by vehicle pre-treated lung slices.

### Ex vivo* bronchodilator studies*

The OA-sensitized animals were sacrificed by experimental concussion followed by rapid exsanguinations. The trachea was removed from the larynx to the bronchi and rapidly placed in a Krebs–Henseleit (KH) solution (NaCl (117.50 mM), KCl (5.60 mM), MgSO_4_ (1.18 mM), CaCl_2_ (2.50 mM), NaH_2_PO_4_ (1.28 mM), NaHCO_3_ (25.0 mM) and D-glucose (5.50 mM), pH = 7.4) at 37 °C, gassed with 95% O_2_ and 5% CO_2_. Using surgical wire, single guinea pig open-ring tracheal strips were connected to an isometric force–displacement transducer (Grass FT03) and the resting tension was adjusted to 0.5 g. After a 60 min equilibration period with three washes, strips were preconstricted with 20 and 40 mM KCl, followed by maximal relaxation established by the addition of (-)-isoproterenol (0.1 µM). After three additional washouts, tracheal preparations were pre-contracted with OA (0.1 μg.ml^−1^) or histamine (1 μM). Cumulative concentration–response curves were constructed using BI01305834 (0.001–10 μM). After washout, basal tone was re-assessed using isoproterenol (10 μM). Tension on tracheal strips was calculated as percentage of 40 mM KCl-induced constriction and expressed as percentage of preconstricted state.

### Randomization and blinding

Animals and slices were randomly assigned to different treatments. The experimenter could not be blinded as animals were individually treated with saline or OA and the solubility of the antagonist was limited. Data analysis and histamine determination was done in a blinded manner.

### Data and analysis

Data are presented as mean ± S.E.M. Data was only subjected to statistical analysis with a minimum group size of n = 5 separate experiments or animals.

All declared group sizes are the number of independent values, statistical analysis was done using these independent values. Outliers were included in data presentation and analysis. The data were checked for normality using D’Agostino’s K-squared test. For in vivo data normality could not be demonstrated and thus a non-parametric approach was used. Statistical evaluation of differences of in vivo data was performed using a Mann–Whitney U test or One-way nonparametric Kruskal–Wallis ANOVA with Dunnett’s post-hoc test where appropriate compared to saline and OA control groups. In order to reduce unwanted sources of variation, data of ex vivo airway narrowing and relaxation studies were normalized to initial airway size and preconstriction, respectively. Histamine determinations were normalized to control. Statistical evaluation of differences of ex vivo data was performed using One- or Two-way ANOVA with Dunnett’s post-hoc test where appropriate. Post-hoc tests were run only if F achieved P < 0.05 and there was no significant variance inhomogeneity. Differences were considered to be statistically significant when p < 0.05. Statistical analysis was performed with GraphPad Prism 5.0 software or Sigmaplot 13 for PCLS dose–response curves.

## Results

### Potency and selectivity data

First the selectivity and potency of the novel TRPA1 antagonist was tested. BI01305834 has a half maximal inhibitory concentration (IC_50_) of 40 nM in human recombinant TRPA1 HEK cells using Ca^2+^ flux as an endpoint and supercinnamaldehyde as the TRPA1 agonist. As shown in Table 2, IC_50_ values against TRPV1, TRPV4, TRPM8 and TRPC6 were all greater than 10 µM, suggesting an excellent selectivity against structurally related TRP channels.

**Table 2:** Analysis of BI01305834 specificity for human TRP-channels

	Potency (IC_50_)	Selectivity vs TRPA1
TRPA1	0.05 µM	na
TRPV1	> 10 µM	> 200
TRPV3	Nd	na
TRPV4	> 10 µM	> 200
TRPC6	> 10 µM	> 200
TRPM8	> 10 µM	> 200

*TRPA* Transient Receptor Potential Ankyrin, *TRPV* Transient Receptor Potential Vanilloid, *TRPC* Transient Receptor Potential Canonical, *TRPM* Transient Receptor Potential Melastatin, *Nd* not determined, *na* not applicable

**Fig. 2:**
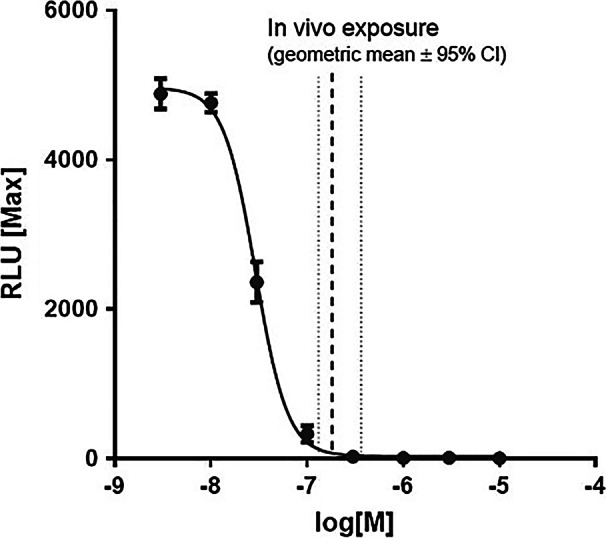
Inhibition of supercinnamaldehyde induced TRPA1 dependent Ca^2^^+^-influx by BI01305834 in guinea pig recombinant TRPA1 HEK cells and in vivo exposure dose. Taking into account the plasma protein binding, the free fraction at time of OA challenge was calculated to be 244 ± 50 nM

BI01305834 showed a similar potency against recombinant guinea pig TRPA1 in HEK cells (IC_50_ = 40 nM) using supercinnamaldehyde as an agonist (Fig. 2). Plasma protein binding in guinea pig was 65%. Clearance following intravenous administration was 10 mL.min^−1^.kg^−1^ and following an oral administration of 1 µmol.kg^−1^ displayed a T_max_ of 1.7 h and an AUC of 508 nM*h. Taken together, BI01305834 displays suitable characteristics for use as a tool compound for in vivo assessment.

### Impact of compound dose on allergen-induced changes in lung function

**Fig. 3:**
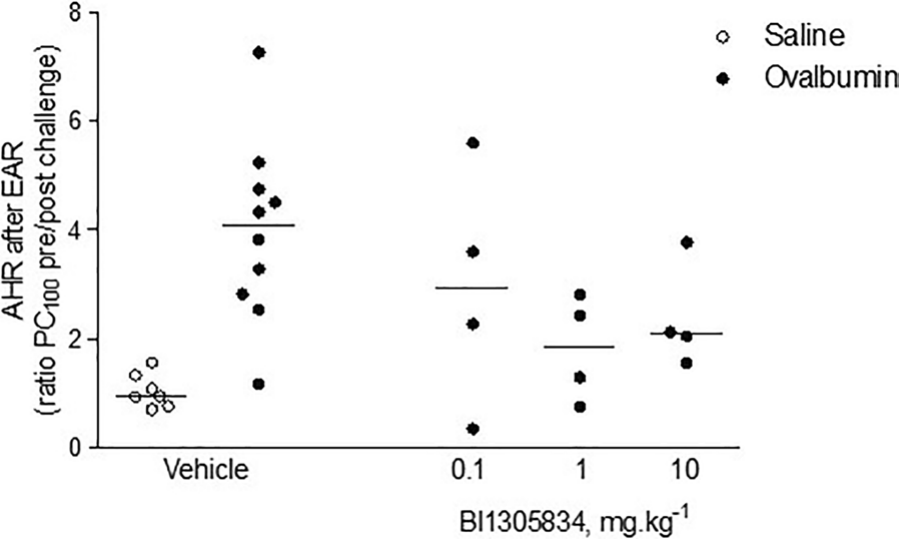
The impact of TRPA1 antagonist treatment in 0.1, 1 and 10 mg.kg^−1^ BI01305834 doses on airway hyperreactivity (AHR) in response to histamine, defined as PC_100pre_/PC_100post_, at t = 6 h, i.e. after the early asthmatic response (EAR). (Veh/Sal = 7 animals, Veh/OA = 10 animals, 0.1/OA = 4 animals, 1/OA = 4 animals, 10/OA = 4 animals)

To study the impact of the TRPA1 antagonist BI01305834 on allergen-induced changes in lung function and inflammation, an in vivo guinea-pig model of acute allergic asthma was used. The primary read-out parameter for efficacy in the pilot study was protection against allergen-induced AHR, measured in response to histamine, around 6 h after ovalbumin exposure, meaning after the EAR. As depicted in Fig. 3, BI01305834 dose-dependently protected against allergen-induced AHR, with 1 mg.kg^−1^ being the most effective dose. Based on these exploratory data, a dose of 1 mg.kg^−1^ was selected for all future studies. The animals used for the pilot experiment were included in the subsequent main study.

Terminal blood samples were taken for measurement of compound concentrations (90 min after compound administration) which on average were 263 ± 53 nM. A concentration–time curve following a dose of 1 µmol.kg^−1^ (comparable to 0.4 mg.kg^−1^) was constructed and the plasma concentrations measured in the experiment extrapolated back to the concentration at time of OA challenge (30 min after compound administration; 368 ± 75 nM). Taking into account the plasma protein binding, the free fraction at time of OA challenge was calculated to be 244 ± 50 nM, which is 6 × guinea pig IC_50_, which is equivalent to IC_99_ in guinea pig (Fig. 2).

### TRPA1 antagonism prevents allergen-induced changes in lung function

**Fig. 4:**
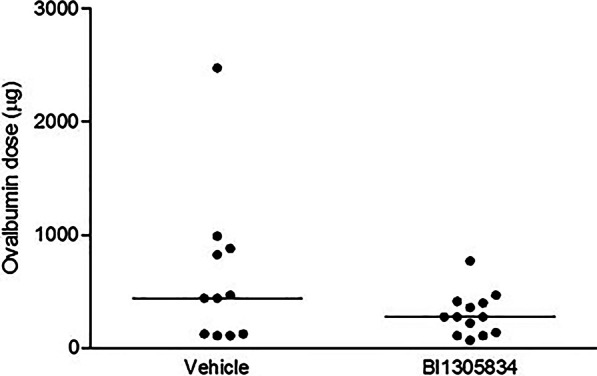
The impact of TRPA1 antagonist treatment (1 mg.kg^−1^ BI01305834) on the dose of ovalbumin (OA) required to induce respiratory distress and an increase in P_pl_ of more than 100%. Animals were exposed to increasing concentrations of ovalbumin and the cumulative dose needed for individual animals is plotted, *P* = 0.17 (Mann–Whitney U test). (Veh = 11 animals, BI = 13 animals)

In the subsequent study, first the impact of BI01305834 on the allergen dose required to induce airflow obstruction was evaluated. This dose was calculated as the product of allergen concentration used and time of exposure until obstruction. In the guinea-pig model, allergen dose is titrated to a physiological response, being respiratory distress and an increase in P_pl_ of more than 100%. Accordingly, animals all receive a slightly different dose of allergen and anti-allergic effects, if existent, can be measured in the model. The TRPA1 antagonist had no effect on the allergen dose required for the initial airflow obstruction (Fig. 4), indicating no direct anti-allergic effect of the compound.

**Fig. 5:**
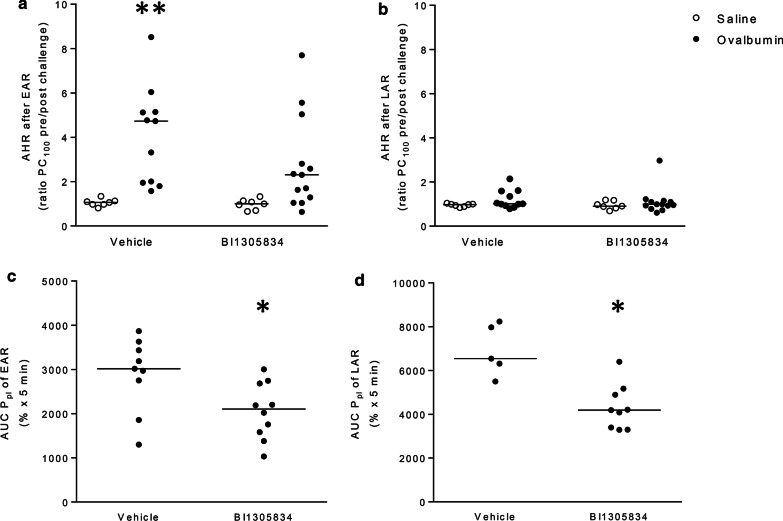
Effect of TRPA1 antagonist (1 mg.kg^−1^ BI01305834) on allergen-induced changes in airway hyperresponsiveness (AHR) and magnitude of the early (EAR) and late asthmatic reaction (LAR). AHR in response to histamine is assessed 6 h after ovalbumin exposure, directly after the EAR (**a**), and 24 h after allergen exposure, directly after the LAR (**b**). Furthermore, the magnitude of the EAR (**c**) and LAR (**d**) were assessed, by calculating the area under the curve (AUC) of the pleural pressure data. *P < 0.05 compared to saline-challenged; vehicle-treated (Kruskal–Wallis ANOVA on ranks followed by Dunnett’s post hoc test), *P < 0.05 (Mann–Whitney *U* test). (Veh/Sal = 7 animals, Veh/OA = 11 animals, BI/Sal = 7 animals, BI/OA = 13 animals)

To study the effect of TRPA1 antagonism on allergen-induced changes in lung function, PC_100_-values were assessed, and AHR in response to histamine was calculated, similar to the pilot study. No significant differences in histamine responsiveness were observed among the groups prior to allergen exposure (data not shown). AHR after the EAR and LAR for each animal is shown in Fig. 5a, b. After the EAR, a 3.9-fold increase in AHR was observed in OA-challenged animals (p < 0.05) (Fig. 5a). Values for the BI01305834-treated control group were not significantly different from vehicle-treated animals. Furthermore, no significant increase in AHR was observed in OA-challenged BI01305834-treated animals. In OA-challenged animals, treatment with BI01305834 resulted in a 1.5-fold decrease in AHR compared to vehicle-treated animals, this was, however, not significant. No AHR was observed directly after the LAR in all groups (Fig. 5b).

In addition to the airway responsiveness to histamine, the magnitude of the EAR and LAR was quantified, by assessing the area under the curve of the pleural pressure curve over time. In response to allergen exposure, a clear EAR and LAR could be recorded in the majority of animals (Fig. 5c, d). Some animals were either disconnected during the measurement, in particular during the overnight measurement, or could not be connected at all as the cannula was gnawn through. For the EAR two animals of the vehicle group and three animals of the BI01305834 group could not be included in the analyses. For the LAR, six and four animals of the vehicle and BI01305834 groups, respectively, had to be excluded from analysis. BI01305834 treatment reduced the size of both the EAR (Fig. 5c) and LAR (Fig. 5a) significantly, 1.4 and 1.6-fold decrease respectively, compared to vehicle-treated animals (p < 0.05).

### TRPA1 antagonism effects on allergen-induced changes in inflammation

**Fig. 6:**
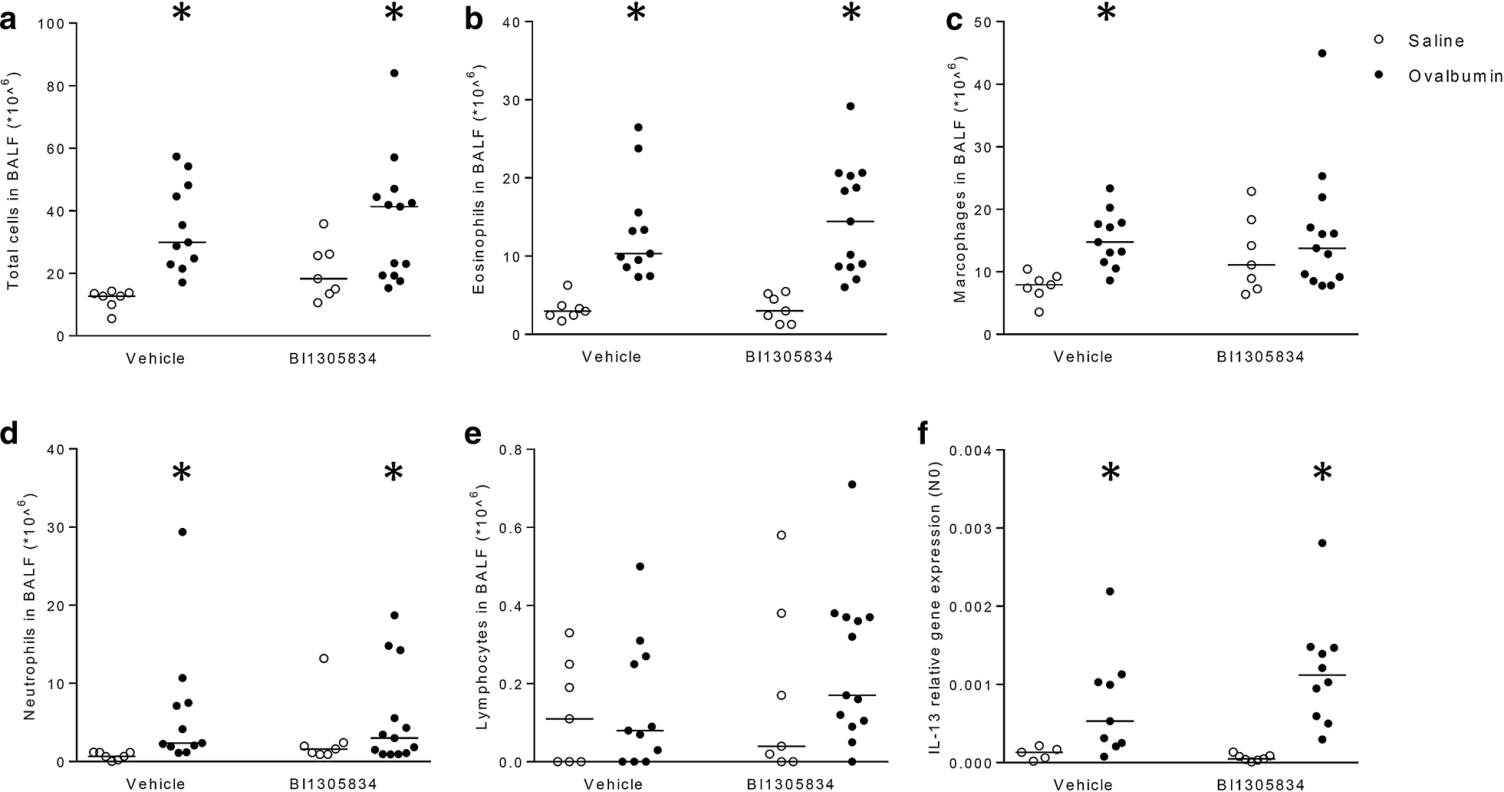
Effect of TRPA1 antagonist (1 mg.kg^−1^ BI01305834) on the allergen-induced changes in total cell number (**a**), eosinophils (**b**); macrophages (**c**); neutrophils (**d**) and lymphocytes (**e**) in the bronchoalveolar lavage fluid (BALF) and on IL-13 mRNA expression in the lung (**f**). *P < 0.05 compared to saline-challenged; vehicle-treated (Kruskal–Wallis ANOVA on ranks followed by Dunnett’s post hoc test) in the bronchoalveolar lavage fluid. (Veh/Sal = 7 animals, Veh/OA = 11 animals, BI/Sal = 7 animals, BI/OA = 13 animals)

After lung function recordings, animals were lavaged to study allergen-induced changes in inflammatory cell infiltration. As shown in Fig. 6a a significant increase in total cell number was observed in the bronchoalveolar lavage fluid from OA challenged animals (p < 0.05). Differential cell analysis showed an increase in eosinophils (p < 0.05) (Fig. 6b), macrophages (p < 0.05) (Fig. 6c) and neutrophils (p < 0.05) (Fig. 6d), with no significant increase in lymphocytes (Fig. 6e) after OA exposure. Comparable increases in inflammatory cells were observed in BI01305834-treated OA-challenged animals; with no statistically significant differences between the vehicle and the BI01305834 treated animals. In subsequent analyses, lung mRNA expression levels of IL-4, IL-5 and IL-13 were assessed. No increase in IL-4 or IL-5 gene expression was observed in OA-challenged animals (data not shown). For IL-13, due to undetectable mRNA levels, two animals of the Veh/Fz and Veh/OA groups, and 3 animals of the BI01305834/OA group had to be excluded from analysis. A significant increase in IL-13 gene expression was observed in OA-challenged animals (Fig. 6f). A comparable increase in TRPA1 antagonist-treated animals was observed, in line with data on inflammatory cells in the lavage fluid. Together, this indicates that in contrast to the lung function data, TRPA1 antagonism had no effect on allergen-induced airway inflammation.

### *TRPA1 antagonism inhibits allergen-induced airway narrowing but not histamine release *in vitro

**Fig. 7:**
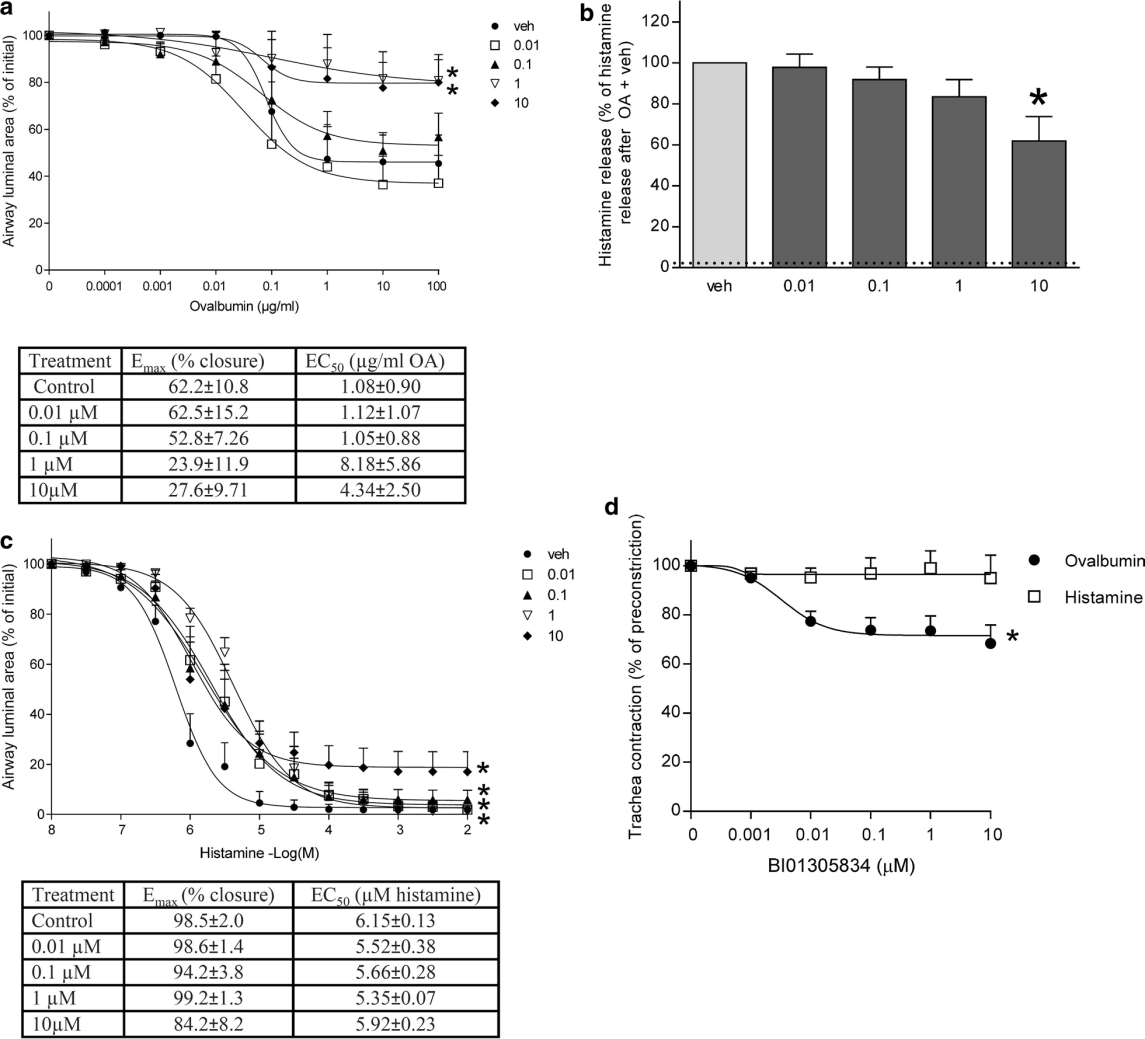
Effect of TRPA1 antagonism with BI01305834 on allergen-induced airway narrowing (**a**), histamine release (**b**) or exogenous histamine-induced airway narrowing (**c**) in vehicle (veh, 10% cyclodextrin) or TRPA1 antagonist (0.01, 0.1, 1 or 10 µM BI01305834) pretreated lung slices of ovalbumin sensitized guinea pigs. Histamine release was calculated as % of total histamine level in both slice and supernatant. Dotted line indicates spontaneous release. Data represents mean ± SEM. *p < 0.05 compared to vehicle (One-(histamine release) or Two-way (airway narrowing) ANOVA with Dunnett’s multiple comparison test). Also, the bronchodilating effect of the TRPA1 antagonist BI01305834 on allergen- and histamine-induced preconstricted tracheal strips of ovalbumin sensitized guinea pigs (**d**). Data represents mean ± SEM. *p < 0.05 One-way ANOVA F-test. (n = 5 animals). Veh, vehicle

As an effect of TRPA1 antagonism on the AHR, EAR and LAR was observed in the guinea pig in vivo model of allergic asthma, we set out to further investigate this mechanism using lung slices and tracheal strips of allergen sensitized guinea pigs. The lung slice model can be used to study functional small airway responses in an intact lung microenvironment [41, 42]. Furthermore, it shows similarity to the in vivo model, as in both models the OA-induced allergic response, i.e. airway narrowing in the lung slice and EAR in the in vivo model, can be inhibited by blocking the histamine H1-receptor [42, 43]. Vehicle or BI01305834 pre-treated lung slices were used to establish allergen dose response curves. As shown in Fig. 7a, stimulation with increasing concentrations of OA results in airway contraction in the sensitized lung slices. Treatment with 1 and 10 µM BI01305834 resulted in a significant suppression of the dose–response relationship of OA-induced airway narrowing compared to vehicle controls (p < 0.05). Furthermore, changes in maximal effect (E_max_) were significantly different (F-test; p < 0.05). No significant changes in sensitivity (EC_50_) were observed (data not shown).

TRPA1 channels are expressed on mast cells [44] and TRPA1 inhibition may prevent mast cell degranulation [28]. Hence, the effect of TRPA1 antagonism on allergen-induced histamine release, as a major mast cell product, was investigated. Stimulation with OA induced a strong increase in histamine release (Fig. 7b). Compared to vehicle treated OA-challenged lung slices, only pretreatment with 10 µM BI01305834 was able to significantly reduce histamine release after OA challenge (p < 0.05)(Fig. 7b). Furthermore, TRPA1 agonism by allyl isothiocyanate (AITC) was unable to induce airway narrowing or histamine release (data not shown).

Next, we set out to study the effect of TRPA1 antagonism on airway narrowing in lung slices induced by exogenous histamine. Exogenous histamine directly stimulates the airway smooth muscle to contract, thus leading to airway narrowing. Indeed, histamine-induced airway narrowing could be inhibited using BI01305834 in guinea-pig lung slices (Fig. 7c). A significant repression of the dose–response relationship of histamine-induced airway narrowing compared to vehicle controls could be observed for all concentrations BI01305834 (p < 0.05). No changes in E_max_ and EC_50_ were observed (Fig. 7).

Lastly, the bronchodilating effect of BI01305834 was further examined using tracheal strips of allergen sensitized guinea pigs. In contrast to lung slices, in which constricted airways will not re-open until baseline lumen diameter because of the loss of tidal breathing forces, tracheal strips do allow for relaxation studies. First the strips were preconstricted using OA or histamine, then a dose response curve of BI01305834 was performed to test the bronchodilating properties of BI01305834. As can be appreciated from Fig. 7d, the TRPA1 antagonist was able to significantly relax OA-preconstricted trachea strips, but not strips preconstricted with histamine (One-way ANOVA, F-test p < 0.05).

## Discussion

The current study aimed to evaluate the efficacy of the novel TRPA1 antagonist BI01305834 in guinea-pig models of allergic asthma. In the in vivo studies, we demonstrate that TRPA1 antagonism with BI01305834 protects against allergen-induced AHR after the EAR (Fig. 5a), and against the development of the EAR (Fig. 5c) and LAR (Fig. 5d). TRPA1 antagonism did not inhibit allergen-induced airway inflammation. The results obtained from the lung slice experiments show that TRPA1 antagonism protects against both allergen and exogenous histamine-induced airway reactions, as is in line with our observations in the in vivo model. Furthermore, using tracheal strips, the bronchodilating properties of BI01305834 in response to allergen-induced constriction were confirmed. Together, this suggests that AHR, EAR and LAR are inhibited by TRPA1 antagonism irrespective of the inflammatory response in a partially histamine-dependent manner.

The in vivo guinea-pig model of allergic asthma used in this study offers a unique chance to test the efficacy of BI01305834 in a whole organism. In airway pharmacology perspective, guinea pigs are superior experimental animals compared to mice and rat, as they better resemble human airway physiology [32]. The specific model used in this study allows for measurements on conscious, unrestrained animals and importantly, it also enables the monitoring of the full EAR and LAR [33]. In the current study, the TRPA1 antagonist BI01305834 was able to reduce the AHR after the EAR. Unfortunately, no AHR after the LAR was observed in the current model, and the effect of BI01305834 on AHR after the LAR could therefore not be evaluated. However, the magnitude of the EAR and LAR themselves was reduced in the presence of protective effect of BI01305834. This is in line with previously reported findings by Raemdonck et al. showing that allergen challenge leads to activation of TRPA1 channels on sensory nerves during the LAR in rats, which resulted in enhanced cholinergic reflex bronchoconstriction [23]. These results are further supported by the fact that in guinea pigs the anticholinergic tiotropium was also able to reduce the EAR and LAR without affecting inflammatory cell infiltration in the BAL [35].

TRPA1 antagonists have been shown to alleviate asthma symptoms in different animal models of asthma. The novel TRPA1 antagonist HC030031 was shown to improve epithelial barrier integrity in a toluene diisocyanate-induced model of occupational asthma [45]. Furthermore, in OA-induced asthma models in mice and rat, HC030031 was able diminish the LAR [23] and reverse the AHR to acetylcholine, albeit at relatively high concentrations [24]. In vitro*-*potency assays show that BI01305834, the TRPA1 antagonist used in this study, is a more potent inhibitor of TRPA1-mediated calcium flux than HC030031, demonstrated by IC_50_-values for TRPA1 of 0.05 µM and 6.2–7.5 µM, respectively [46, 47].

As a second aim, we investigated how TRPA1 antagonism could alleviate asthma symptoms in guinea-pig models of allergic asthma. Lung slices of allergen-sensitized guinea pig were used, as they allow for investigation of functional airway responses in the lung tissue [42]. In contrast to the in vivo model, the lung microenvironment represented by the lung slice model enabled us to study the effect of TRPA1 inhibition on airway smooth muscle contraction in a more direct manner, as well as the contribution of mast cells. An additional advantage of the lung slices is that many lung slices can be obtained from one animal, and therefore multiple conditions can be tested in the same animal [41]. The results obtained in the OA-induced experiments confirmed the protective and bronchodilating effect of BI01305834 observed in vivo*,* in particular on OA-induced airway narrowing, and in smaller extent in the pre-constricted trachea strips. More modest inhibitory effects were seen on exogenous histamine-induced airway narrowing in lung slices, with no inhibitory effect on histamine pre-constricted strips. The exogenous histamine used in the lung slices induces airway narrowing by directly affecting the airway smooth muscle. In contrast, the OA-induced airway narrowing is based on the release of contractile mediators in lower concentrations in response to allergen provocation, by for example mast cells. These mediators will in turn affect the contractility of the airway smooth muscle. As the allergen-induced smooth muscle contractility is a secondary effect of OA-provocation in the lung slice, this could explain why a relatively smaller maximal effect in airway narrowing is induced by OA in comparison to the full airway closure that is induced by exogenous histamine. Another study in our lab showed similar difference in maximal airway closure induced by OA and histamine [48]. Furthermore, the mix of mediators released from the mast cell includes not only histamine, but also serotonin, TxA2 and cysteinyl leukotrienes, all of which have been shown to contribute to the functional airway narrowing response [42, 43]. AITC was unable to induce airway narrowing or histamine release in the lung slices. Similar findings were reported in a mouse model of chemical induced asthma, where AITC was unable to induce AHR in toluene-2,4-diisocyanate-sensitized mice while TRPA1 blockage or knock-out prevented the development of AHR [49]. Together this suggests that TRPA1 agonism by itself is not enough to induce bronchoconstriction, whereas TRPA1 antagonism is able to prevent allergen-induced airway narrowing.

The mast cell is an important player in allergen-induced airway narrowing, and an inflammatory cell type key to asthma pathophysiology. Upon antigen binding to their surface receptors mast cells release pro-contractile mediators including histamine, thereby inducing airway smooth muscle contraction [50]. Interestingly, mast cells express TRPA1 channels [44] and are able to induce sensitization of sensory nerves [27]. Furthermore, TRPA1 affects mast cell degranulation [51]. In the lung slices, we observed a small decrease in histamine release by the highest concentration antagonist that may play a role in the protective effect of BI01305834. However, it does not seem likely that mast cell-mediated effects and especially allergen-induced histamine release by mast cells, can fully explain the alleviation of asthma symptoms after TRPA1 antagonism as observed in vivo*.* As we observed only small effects and these results do not completely comply with the results obtained in the OA-induced airway narrowing experiment where 1 µM of BI01305834 was already able to achieve maximal protection. In the in vivo study, the free fraction of BI01305834 was calculated to be 244 ± 50 nM at time of OA challenge after administration of 1 mg.kg^−1^ BI01305834. In the lung slice model concentrations of 0.01, 0.1, 1 and 10 µM BI01305834 were used. This means that the results obtained with 10 µM BI01305834 in the lung slices are supraphysiological compared with the effects seen in vivo. As we measured the IC_50_ for cytotoxicity in U937 cells to be 640 µM, we do not expect the observed results on histamine-induced airway narrowing and histamine release after administration of 10 µM BI01305834 are related to toxicity. Rather, we expect this to be an off-target effect induced by the supraphysiological dose of the compound. This may also explain the lack of effect of AITC on histamine release. In summary, this indicates that the protective effect of BI01305834 on allergen-induced changes in asthma models was probably not mediated via TRPA1 channels on mast cells, as both in vivo and *ex* vivo these effects were observed at lower concentrations already.

We know from previous experiments in our lab that there is no effect of atropine on basal airway tone in the guinea-pig lung slice. Furthermore, spontaneous neurotransmitter release from guinea-pig tracheal preparations (measured by HPLC) is not detectable; electric field stimulation is necessary to achieve such release [52]. This essentially rules out the possibility that neurotransmitter release will interfere in this setting. An explanation for the observed protective effects of BI01305834 as being primarily mediated via TRPA1 channels expressed on nerves, is therefore quite unlikely in this setting. However, as we observed a small, but protective, effect of TRPA1 antagonism on exogenous histamine-induced airway narrowing in the lung slice, this suggests that TRPA1 channels expressed on other cell types in the lung, in particular airway smooth muscle cells, may also contribute to the protective effect of TRPA1 antagonism, and histamine may be involved in this mechanism. Histamine receptor ligation activates, among others, phospholipase C, resulting in the generation of intracellular inositol-(1,4,5)-triphosphate (IP_3_) via the hydrolysis of phosphatidylinositol-(4,5)-biphosphate (PIP_2_) [18, 53]. The release of intracellular calcium induced by IP_3_ is thought to be involved in the activation of TRPA1 [54]. As airway smooth muscle cells express calcium permeable TRPA1 channels [25], and histamine stimulation of smooth muscle results in intracellular calcium peaks required for constriction, this suggests a direct role for TRPA1 in smooth muscle contraction, independent of neuronal contribution. It is possible that also other mediators are involved in the TRPA1-mediated effect on bronchoconstriction. Grace et al*.* suggested a role for prostaglandin E2 (PGE2) and bradykinin in TRPA1-related nerve activation [15]. Furthermore, bradykinin is known to stimulate the release of 15-hydroxyeicosatetraenoic acid (15-HETE) and PGE2 by bronchial epithelial cells [55]. From studies into the role of TRPA1 in pain, it is known that TRPA1, the histamine H1-receptor and 15-HETE, work synergistically to induce nociception [56]. This suggests a direct role for TRPA1 in smooth muscle contraction, independent of neuronal contribution.

Inflammation is an important part of asthma pathology, and sensory nerve activation may also contribute to this by inducing neurogenic inflammation [15]. Against expectations, and in contrast with current literature, we did not observe an anti-inflammatory effect of TRPA1 antagonism in our in vivo guinea-pig model. Thus, BI01305834 did not affect inflammatory cells in the lavage fluid, or IL-13 gene expression in lung homogenates. Substance P and neurokinin A were measured in the lavage fluid as mediators of neurogenic inflammation, but were below detection limit (data not shown). In part, the fact that TRPA1 antagonism did not affect inflammation in the current study, might be explained by the different allergen exposure protocols that were used in the animal models. Caceres et al. challenged mice for three consecutive days [24], whereas the guinea pigs in our study were challenged only once. Previously, it was shown that in an in vivo guinea-pig model of chronic asthma, multiple allergen challenges will result in a more developed allergic response, possibly resulting in differential outcomes after similar treatments, as was shown for budesonide and tiotropium in the current model [57]. Both were not effective in inhibiting inflammation in the acute model, but do inhibit inflammation in the chronic model, using twelve OA challenges as compared to one OA challenge in the acute model [58]. Testing BI01305834 in a chronic asthma model might therefore be a more suitable way to test its effect on airway inflammation. Nevertheless, it is also possible that BI01305834 inhibits AHR and allergen-induced asthmatic responses without effecting inflammation.

Airway inflammation is important for the development of AHR [59]. However, other mechanisms are also involved, as AHR persists in the absence of inflammation. Here, there might be an important role for airway nerves, as vagotomy or anticholinergic treatment inhibits AHR in experimental models [26, 43, 60]. It is increasingly recognized that neural plasticity and increased nerve activity contribute to many symptoms of asthma including AHR and cough [11]. Both animal studies and human biopsy studies suggest that the airway neural network is more dense in asthma [12, 13, 61]. Enhanced activity of sensory nerves, via increased excitability or lowering of activation threshold and enhanced transmission, may further contribute to asthma symptoms [62]. For example, OA challenge in rats induces hypersensitivity of pulmonary C-fibers, with increased baseline activity and an increased response to the TRPV1 agonist capsaicin [63]. Together, this further supports nerve targeting approaches such as TRPA1 antagonism as a treatment for asthma.

Currently, there are no clinically approved TRPA1 antagonist available. However, many preclinical studies show the potential for TRPA1 antagonism in asthma and other airway disorders, including chronic obstructive pulmonary disease and cough [30]. Based on the results reported in this study, BI01305834 shows to be an effective bronchodilator in allergen-induced asthmatic reactions. Existing bronchodilators are β-adrenoreceptors agonists, muscarinic acetylcholine receptor antagonists or xanthines [64]. Although they improve the quality of life of many patients, most of the times, the usage of current bronchodilators does not lead to full symptom relief. Interestingly, TRPA1 antagonists are a good potential additional treatment option, as they combine bronchoprotective activity and antitussive activity, and may have anti-inflammatory effects as well. LABAs and tiotropium most certainly are good bronchoprotectors, but have limited effects on cough. The anti-inflammatory effects of LABAs are limited [65], whereas anti-inflammatory effects of LAMAs have been noted in preclinical models [66, 67], but are still under investigation in the clinical setting. Thus, alleviation of both bronchoconstriction, inflammation and cough by a single target may be of benefit for patients with severe asthma. Furthermore, TRPA1 antagonist may be beneficial in treatment-resistant and non-allergic asthma as well, as cough responses are shown to be unrelated to AHR, airflow obstruction and treatment with inhaled corticosteroid [68].

## Conclusions

In conclusion, our study shows that the novel antagonist BI01305834 is a potent and selective TRPA1 inhibitor that reduces airway hyperresponsiveness and the early and late asthmatic response in vivo. Also, in the ex vivo systems of precision-cut lung slices and isolated tracheal strips of sensitized guinea pigs, TRPA1 antagonism was able to protect against allergen and histamine-induced airway narrowing, and reverse allergen-induced bronchoconstriction, respectively. BI01305834 seems to alleviate asthma symptoms via TRPA1 channels expressed on sensory nerves, however, TRPA1 expressed on other cells types may enhance this protective effect. These findings show that TRPA1 makes an interesting target for asthma therapy.

## Data Availability

All data generated or analysed during this study are included in this published article. BI01305834 is developed by Boehringer Ingelheim. Boehringer Ingelheim supports research collaborations surrounding BI01305834.

## Abbreviations

15-HETE: 15-Hydroxyeicosatetraenoic acid
AHR: Airway hyperresponsiveness
AITC: Allyl isothiocyanate
EAR: Early asthmatic reaction
EC_50_: Allergen sensitivity
Emax: Maximal effect
IC_50_: Half maximal inhibitory concentration
IgE: Immunoglobulin E
IL: Interleukin
LAR: Late asthmatic reaction
OA: Ovalbumin
PGE2: Prostaglandin E2
P_pl_: Pleural pressure
TRPA1: Transient Receptor Potential Ankyrin 1
